# Deletion of exon 2 in ALS-linked *Sptlc1* causes lethality in homozygous mice but not in heterozygotes

**DOI:** 10.26508/lsa.202503605

**Published:** 2026-07-02

**Authors:** Devesh C Pant, Museer A Lone, Janani Parameswaran, Fuying Ma, Nicole Ziak, Prisha Dutta, Zitong Wang, Daniel Pun, Sumit Verma, Thorsten Hornemann, Jie Jiang

**Affiliations:** 1 Department of Cell Biology, Emory University , Atlanta, GA, USA; 2 Institute for Clinical Chemistry, University of Zurich , Zurich, Switzerland; 3 Department of Pediatrics and Neurology, Emory University , Atlanta, GA, USA

## Abstract

Mutations in the human *SPTLC1* gene have recently been linked to early-onset amyotrophic lateral sclerosis (ALS), characterized by global atrophy, motor impairments, and symptoms such as tongue fasciculations. All known ALS-linked *SPTLC1* mutations cluster within exon 2, and a specific variant, c.58G>T, results in exon 2 skipping. However, it is unclear how the exon 2 deletion affects SPTLC1 function in vivo and contributes to ALS pathogenesis. Leveraging the high genomic sequence similarity between mouse and human *SPTLC1*, we created a novel knock-in mouse model with a CRISPR/Cas9-mediated deletion of exon 2 in the endogenous murine *Sptlc1* locus. Although heterozygous mice did not develop motor defects or ALS-like neuropathology, homozygous mutants died prematurely. These findings provide valuable insights into SPTLC1 exon 2 biology and serve as a useful resource for future mechanistic studies.

## Abstract (precis)

Pant et al generate a knock-in mouse model deleting exon 2 of Sptlc1, a hotspot for ALS-linked mutations. Homozygous deletion causes early lethality, whereas heterozygous mice show no motor or neuropathological abnormalities, highlighting distinct dosage-dependent roles of Sptlc1 in vivo.

## Introduction

Early-onset ALS, also known as juvenile ALS (jALS), is a rare subtype of ALS that affects individuals younger than 25. Although ALS primarily manifests in adults aged 50–70, studying jALS can provide valuable insights into ALS pathogenesis, as both forms share the hallmark degeneration of upper and lower motor neurons. However, the clinical heterogeneity of jALS poses significant challenges for diagnosis and management, with many patients remaining undiagnosed and the underlying causes of their symptoms elusive. Advances in sequencing technologies have uncovered novel genetic defects in ALS that were previously unrecognized as contributors to neurological diseases (1). Recently, *SPTLC1* (#605712; OMIM) variants have been reported in jALS cohorts across European and Asian populations (2, 3, 4, 5, 6), joining a growing list of genes associated with jALS, including *FUS*, *SETX*, and *ALS2*. Electrophysiological and histopathological analyses of muscle and nerve biopsies from SPTLC1-jALS patients reveal diffuse acute and chronic denervation in multiple myotomes without sensory neuropathy. Notably, mild cognitive dysfunction has been reported in two SPTLC1-jALS patients, reinforcing the idea that ALS forms a disease spectrum with dementia (3).

*SPTLC1* is a highly conserved gene that encodes a subunit of serine palmitoyltransferase (SPT), the enzyme catalyzing the rate-limiting first step in sphingolipid biosynthesis. Sphingolipids are critical components of cell membranes and signaling pathways, and their involvement in central nervous system (CNS) disorders has been discussed in our review (7). The SPT complex, a multi-subunit enzyme, conjugates palmitoyl-CoA and L-serine to produce long-chain bases (LCBs), the precursors of sphingolipids. The SPT enzyme complex resides in the ER membrane and at the ER–mitochondrion contact sites. In mammals, the enzyme consists of two of the three primary subunits, SPTLC1/SPTLC2 or SPTLC1/SPTLC3, that assemble into tetrameric complexes (8, 9). Mutations in the C terminus of *SPTLC1* have been previously linked to hereditary sensory and autonomic neuropathy type 1 (HSAN1), a group of rare peripheral neuropathies characterized by the formation of noncanonical deoxysphingolipids (10, 11). In contrast, ALS-associated *SPTLC1* variants are primarily found near the transmembrane (TM) interface encoded by exon 2. One specific variant (*SPTLC1* c.58 G >T) results in a single base-pair change adjacent to the splice acceptor site for exon 2. The c.58G>T variant predominantly leads to an in-frame deletion of amino acids 20–56 comprising the entire TM domain.

The *SPTLC1* gene is ubiquitously expressed and initiates de novo sphingolipid synthesis. *SPTLC1*-linked HSAN1 mutation (C133W) has been previously studied in mice using transgenic or knock-in approaches. The transgenic mice were reported to display a mild phenotype. However, the knock-in heterozygotes were reported to be normal and homozygotes died embryonically (12, 13, 14). To date, there are no published murine models for SPTLC1-related ALS. The current evidence surrounding sphingolipid profiles in SPTLC1 ΔExon2 ALS human patients remains sparse. One study indicated an increase in serum sphingolipids (5), whereas another group reported no changes in plasma ceramide or sphingomyelin levels in patients carrying the exon 2 skipping variant (3). Notably, blood lipids can vary considerably within individuals over short periods of time because of intrinsic factors, such as hormonal variation or diet (15). Given the very small sample sizes in the original studies, it is difficult to draw meaningful insights from these data or from follow-up overexpression studies using cell lines (3, 5, 16).

In this study, we generated *Sptlc1* exon 2 deletion (ΔE2) knock-in mice to investigate the pathogenic mechanisms of *SPTLC1*-linked ALS. Mice lacking *Sptlc1* exon 2 in both alleles exhibited embryonic lethality with incomplete penetrance. Longitudinal motor function assessments revealed that the SPTLC1 ΔE2 mutation did not induce an ALS-like phenotype in heterozygous mice, nor did it alter canonical sphingolipid levels. Furthermore, neuropathological analysis showed no changes in markers of neuroinflammation, such as astrogliosis or microglial activation. Our findings provide new insights into the in vivo roles of SPTLC1 ΔE2 mutation and suggest that the ΔE2 heterozygous mice do not develop overt motor deficits or gross neuropathological changes up to 18 mo of age under standard housing conditions.

## Results

### Generation of first *Sptlc1* exon 2 deletion knock-in mouse model

**Figure 1.**
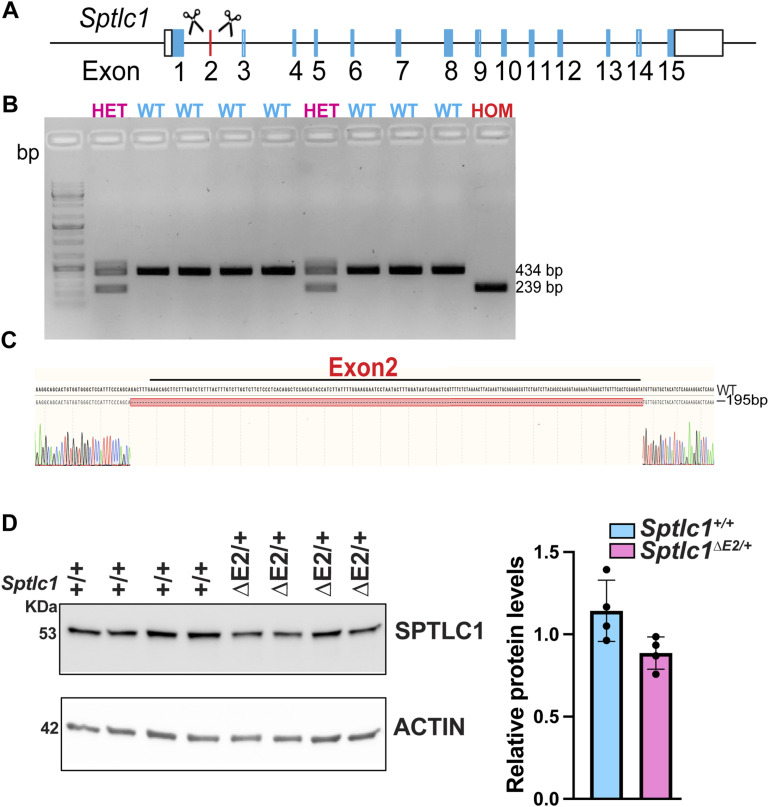
Generation of *Sptlc1* ΔE2 knock-in mice using CRISPR/Cas9 technology. **(A)** Gene diagram of the mouse *Sptlc1* locus showing the location of the exon 2 deletion. **(B)** Agarose gel electrophoresis with genotyping primers demonstrates precise deletion of exon 2 using the CRISPR/Cas9 knock-in approach. The heterozygous (HET) mouse shows two bands: the upper WT and a lower ΔE2 band, whereas the homozygous (HOM) mouse exhibits only the ΔE2 band. **(C)** Sanger sequencing of the purified PCR product from the HET animal confirms the *Sptlc1* WT and exon 2 deletion (−195 bp) sequences. **(D)** Western blot analysis shows no statistically significant difference in total SPTLC1 protein levels between *Sptlc1*^+/+^ and *Sptlc1*^ΔE2/+^ spinal cords. Lysates from n = 4 mice per group were analyzed, and SPTLC1 levels were normalized to ACTIN. Values are the mean ± SD, *t* test, significance threshold set at *P* < 0.05. Source data are available for this figure.

Exon 2 represents the most common mutational hotspot in human *SPTLC1*-associated jALS. To develop an in vivo model that closely mirrors the human condition and to investigate the underlying disease mechanism, we generated the first *Sptlc1* knock-in mouse model harboring an exon 2 deletion (ΔE2) using CRISPR/Cas9 genome editing (Fig 1A). Two founder lines carrying a 195-bp deletion encompassing exon 2 were established. PCR amplification of genomic DNA followed by agarose gel electrophoresis revealed distinct band patterns: heterozygous *Sptlc1*^*ΔE2/+*^ mice had two bands of 434 bp and 239 bp, respectively, with the difference corresponding to the deletion of exon 2; WT *Sptlc1*^*+/+*^ mice displayed a single 434-bp band; and homozygous *Sptlc1*^*ΔE2/ΔE2*^ mice displayed one single band of 239 bp (Fig 1B). Sanger sequencing of the PCR products confirmed the removal of exon 2 in these mice (Fig 1C). Among 100 offspring from 10 independent matings, only three homozygous mutant mice were identified, compared with 33 WT and 64 heterozygous littermates. These three homozygous animals ultimately failed to thrive and died within 6–8 wk of age, suggesting that the homozygous ΔE2 mutation results in embryonic lethality with incomplete penetrance. To assess protein levels, spinal cord tissues were collected from 3-wk-old animals. Western blot analysis showed that heterozygous *Sptlc1*^ΔE2/+^ mice exhibited no statistically significant difference compared with *Sptlc1*^+/+^ littermates (Fig 1D).

### No alteration in canonical sphingolipid levels in *Sptlc1*^*ΔE2/+*^ mice

**Figure 2.**
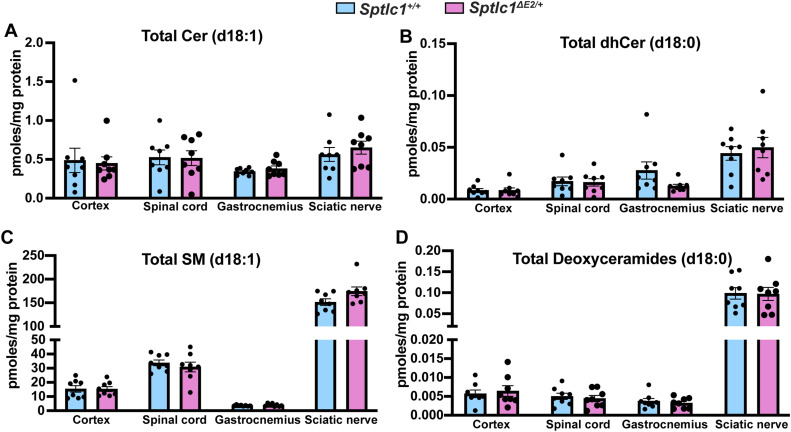
No alterations in lipidomics profile in *Sptlc1*^*ΔE2/+*^ mouse tissues. **(A, B, C, D)** Similar profiles of total ceramides (A), total dihydroceramides (B), total sphingomyelins (C), and total deoxyceramides (D) were observed in different mouse tissues (cortex, spinal cord, gastrocnemius, and sciatic nerve) in *Sptlc1*^ΔE2/+^ compared with *Sptlc1*^+/+^. Data points are individual mice. Values are the mean ± SEM (One-way ANOVA, n = 8 mice per group). Significance threshold set at *P* < 0.05.

SPTLC1 is a core component of the SPT complex, which catalyzes the rate-limiting first step in sphingolipid biosynthesis by conjugating L-serine with palmitoyl-CoA to generate sphingosine, the precursor to ceramide and complex sphingolipids. Given that ALS-associated *SPTLC1* variants have been proposed to disrupt sphingolipid homeostasis, we sought to determine the effect of the exon 2 deletion on total sphingolipid levels in vivo. Using mass spectrometry, we quantified canonical sphingolipids, including ceramides, dihydroceramides, and sphingomyelins in both CNS and peripheral tissues of *Sptlc1*^*ΔE2/+*^ mice and littermate controls at 4 mo of age. Surprisingly, heterozygous *Sptlc1*^*ΔE2/+*^ mice did not exhibit any significant alterations in sphingolipid levels across multiple tissues, including the cortex, spinal cord, gastrocnemius muscle, and sciatic nerve (Fig 2A–C). These findings suggest that the *Sptlc1* ΔE2 mutation does not impair steady-state sphingolipid metabolism under basal conditions. Similarly, the total level of deoxyceramides, which is associated with neurotoxicity in HSAN1, was not changed in heterozygous *Sptlc1*^ΔE2/+^ mice (Fig 2D).

### *Sptlc1*^*ΔE2/+*^ mice do not develop impaired motor function

**Figure 3.**
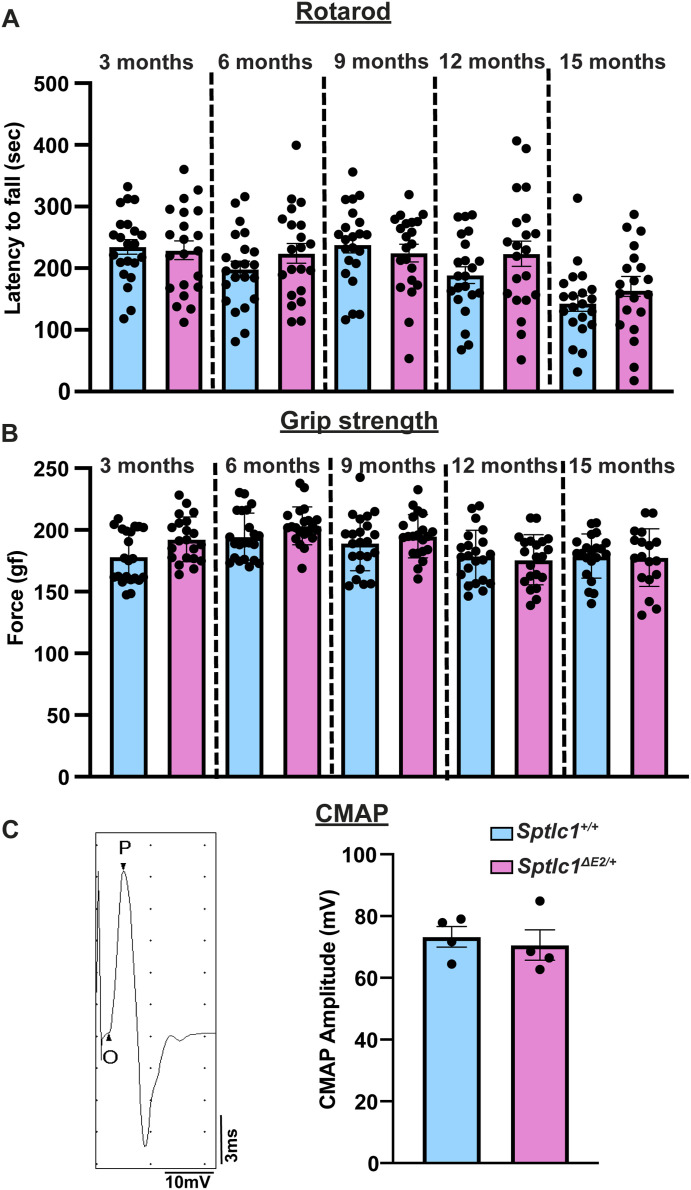
*Sptlc1*^*ΔE2/+*^ mice do not display motor impairments. **(A)** Longitudinal rotarod analysis revealed that *Sptlc1*^ΔE2/+^ mice, both males and females, showed no significant changes compared with *Sptlc1*^+/+^ littermates. **(B)** Muscle strength was assessed using a grip strength test, and *Sptlc1*^ΔE2/+^ mice performed similarly compared with *Sptlc1*^+/+^ (Two-way ANOVA, n = 18–20 mice per group). **(C)** Compound muscle action potential amplitudes in the hind paw muscles, elicited from nerve stimulation at the ankle, showed no differences in 17-mo-old animals (*t* test, n = 4 mice per group). Significance threshold set at *P* < 0.05.

To assess the effects of the ΔE2 mutation on motor function, we evaluated mice using various motor and behavioral tests at different time points. Male and female mice were tested on the rotarod assay beginning at 3 mo of age, with no significant differences observed between *Sptlc1*^*+/+*^ and *Sptlc1*^ΔE2/+^ mice up to 15 mo of age (Fig 3A). To further evaluate motor strength, we conducted grip strength assays through 15 mo of age. No significant differences were detected between groups at any time point, based on the average performance across three trials (Fig 3B). In addition, electrophysiological recordings revealed no differences in compound muscle action potential (CMAP) amplitude in the hindlimb muscles at 17 mo of age (Fig 3C). Overall, these longitudinal behavioral analyses did not identify any motor impairments in *Sptlc1*^*ΔE2/+*^ mice, suggesting that the heterozygous ΔE2 mutation does not lead to overt motor or neuromuscular dysfunction.

### *Sptlc1*^*ΔE2/+*^ mice do not display neuronal loss or gliosis in the CNS

**Figure 4.**
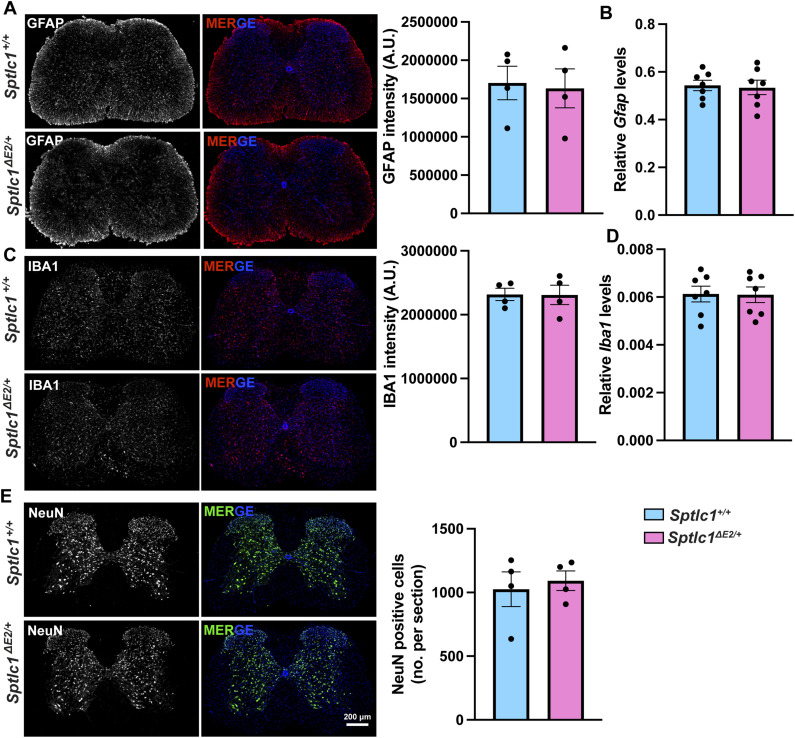
No evidence of reactive gliosis and neuronal loss in the *Sptlc1*^*ΔE2/+*^ mice. **(A)** GFAP immunofluorescence staining in 18-mo-old lumbar spinal cord region. Average GFAP fluorescence intensity quantification is on right (n = 4 mice per group). **(B)** qRT–PCR confirms no significant difference in *Gfap* levels (n = 7 mice per group). **(C)** IBA1 immunofluorescence staining in 18-mo-old lumbar spinal cord region. Average IBA1 fluorescence intensity quantification is on right (n = 4 mice per group). **(D)** qRT–PCR confirms no significant difference in *Iba1* levels (n = 7 mice per group). **(E)** NeuN was used as a neuronal marker, and NeuN-positive cell quantification is on right. DAPI was used to stain nuclei. N = 4 mice per group. Data are presented as the mean ± SEM, *t* test, significance threshold set at *P* < 0.05.

**Figure S1.**
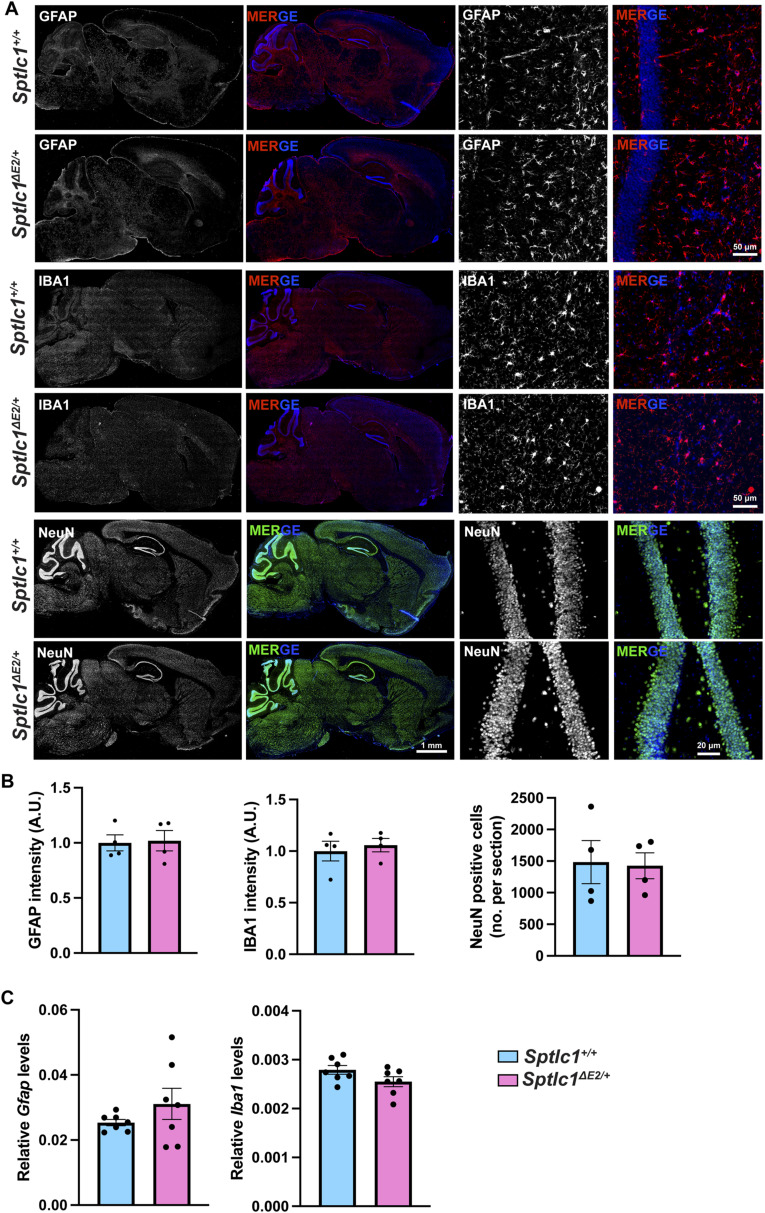
No evidence of reactive gliosis and neuronal loss in the *Sptlc1^ΔE2/^*^+^ mouse brain. **(A)** GFAP and IBA1 immunofluorescence staining in 18-mo-old brain sagittal section. The GFAP and IBA1 signals in *Sptlc1^ΔE2/^*^+^ were compared with WT littermates. Higher magnification images are shown. NeuN was used as a neuronal marker. DAPI was used to stain nuclei. N = 4 mice per group. **(B)** Average fluorescence intensity quantifications of GFAP and IBA1, as well as the number of NeuN-positive cells. **(C)** qRT–PCR confirms no significant difference in *Gfap* or *Iba1* levels in the cortex region of *Sptlc1^ΔE2/^*^+^ compared with controls or *Sptlc*^+/+^ 18-mo-old animals (*P* < 0.05, *t* test, n = 7 mice per group).

We next performed a comprehensive neuropathological analysis to determine the effects of the ΔE2 mutation. Immunofluorescence staining for NeuN, IBA1, and GFAP was performed on sagittal brain and lumbar spinal cord sections from 18-mo-old mice. There were no statistically significant differences in astrocyte or microglial activation, as GFAP and IBA1 levels remained unaltered (Fig 4A–D). In addition, no obvious loss of neurons was observed in either spinal cord (Fig 4E) or brain (Fig S1A–C). Taken together, these findings indicate that heterozygous *Sptlc1*^*ΔE2/+*^ mice do not display any noticeable cellular or molecular indicators of neurodegeneration or glial dysfunction.

### *Sptlc1*^*ΔE2/+*^ mice exhibit no detectable changes in the level of the SPT complex

**Figure 5.**
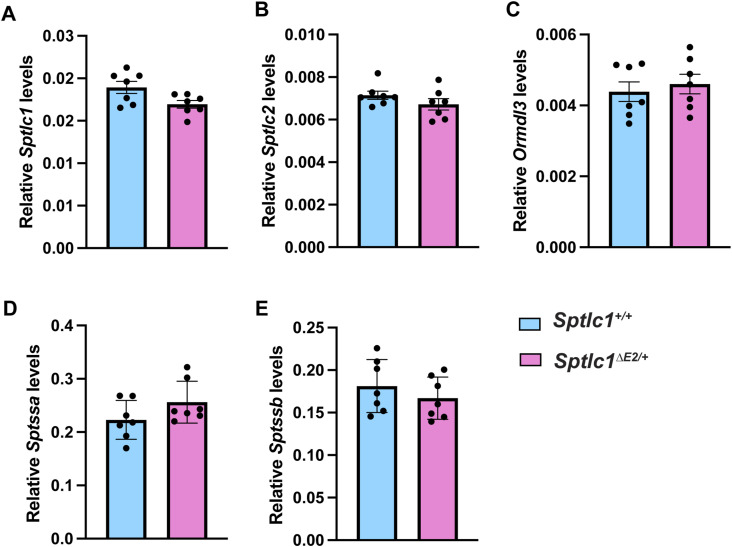
*Sptlc1*^*ΔE2/+*^ did not induce any changes in the SPT-ORMDL complex. **(A)** Total *Sptlc1* mRNA levels were measured using exon 3–4 junction probes. **(B)** Total *Sptlc2* mRNA levels were measured using exon 4–5 junction probes. **(C)** Total *Ormdl3* mRNA levels were measured using exon 4 probes. **(D)** Total *Sptssa* mRNA levels were measured using exon 1–2 junction probes. **(E)** Total *Sptssb* mRNA levels were measured using exon 4 probes. Data are presented as the mean ± SEM, *t* test, significance threshold set at *P* < 0.05. n = 7 mice per group.

Recent structural studies have provided key insights into the organization and regulation of the SPT complex. SPTLC1 and SPTLC2 form the catalytic core of the enzyme, whereas ORMDL proteins act as negative regulators by modulating SPT activity in response to sphingolipid levels (18). Given that *SPTLC1*-linked jALS variants cluster near the transmembrane domain, potentially affecting protein–protein interactions within the complex, we investigated whether the *Sptlc1* ΔE2 mutation alters the expression of key SPT components in vivo. Because of the lack of commercial antibodies for mouse SPTLC2 and ORMDL3, we quantified transcript levels using RT–qPCR. Expression analysis revealed no statistically significant differences in *Sptlc1* (Fig 5A), *Sptlc2* (Fig 5B), *Ormdl3* (Fig 5C), *Sptssa* (Fig 5D), *and Sptssb* (Fig 5E) mRNA levels in *Sptlc1*^*ΔE2/+*^ mice compared with littermate controls. These findings suggest that the *Sptlc1* ΔE2 mutation does not disrupt the transcriptional expression of SPT complex components.

## Discussion

In this study, we generated and characterized a novel *Sptlc1* exon 2 deletion knock-in mouse model to investigate the pathogenic mechanisms of *SPTLC1*-associated jALS. Although prior studies have suggested that *SPTLC1*-jALS variants lead to dysregulated sphingolipid metabolism, our findings indicate that heterozygous (*Sptlc1*^*ΔE2/+*^) mice do not exhibit significant disruptions in sphingolipid homeostasis, motor function, neuroanatomy, or neuropathology.

To date, missense mutations in *SPTLC1* have been linked to several human diseases, including ALS, HSAN1, and Flegel disease (5, 10, 19). HSAN1-linked *SPTLC1* mutations typically result in a loss of substrate specificity, causing the SPT enzyme to incorporate alanine or glycine instead of serine, thereby producing deoxysphingolipids. Deoxysphingolipids lack a critical hydroxyl moiety, cannot be efficiently degraded by cellular machinery, and cause toxicity. In contrast, ALS-associated *SPTLC1* variants, including Y23F, L38R, L39del, and F40S41del, are clustered in exon 2 and one of the variants, c.58G>T, leads to exon 2 skipping (5, 20). Exon 2 of *SPTLC1* encodes a transmembrane domain that interacts with ORMDL proteins, negative regulators of SPT activity. By overexpressing the ALS-associated *SPTLC1* mutations in HEK293 cells with endogenous *SPTLC1* deleted, Lone et al showed that these variants impaired its interaction with ORMDLs, resulting in increased sphingolipid synthesis and a distinct lipid signature (16). Canonical sphingolipids were also shown to increase in human iPSC-derived motor neurons using a compound-heterozygous (*SPTLC1*^*ΔF40_S41/ΔE2*^) line (5). However, ALS patients reported to date do not carry compound-heterozygous mutations. Therefore, an increase in sphingolipids in this model might be driven by a different mechanism.

**Figure S2.**
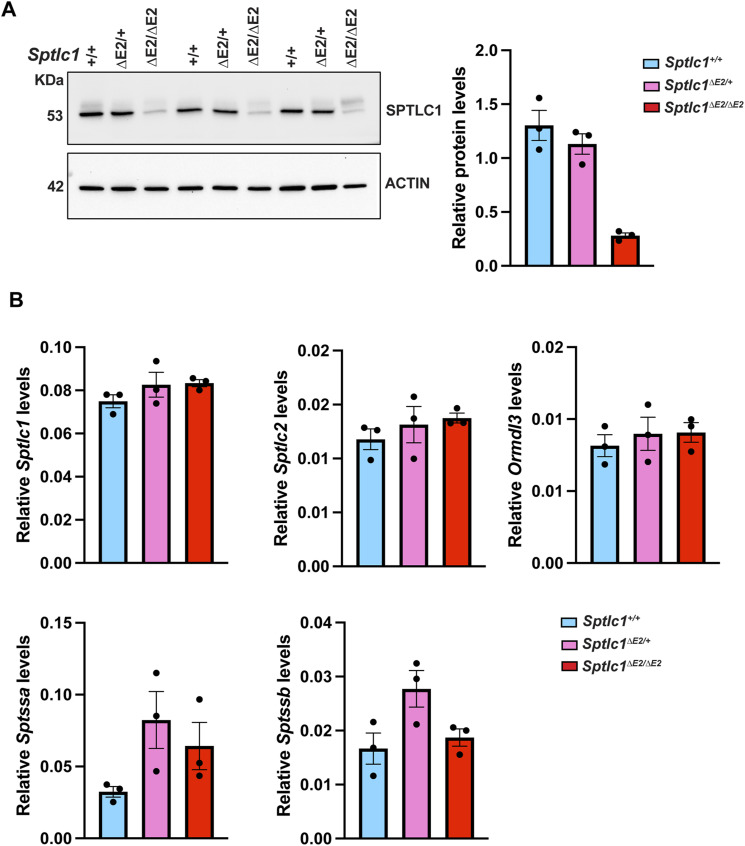
Characterization of SPTLC1 protein levels and the SPT-ORMDL complex at early time point. **(A)** Reduced total SPTLC1 protein levels in the 2-mo-old homo-zygous *Sptlc1^ΔE2/ΔE2^* spinal cord (n = 3). **(B)** No change in the SPT- ORMDL complex at an early time point in the cortex by RT–PCR. Data are presented as the mean ± SEM (n = 3 mice per genotype). Source data are available for this figure.

Despite this in vitro evidence, we found no significant alterations in canonical sphingolipid levels in the CNS or peripheral tissues of *Sptlc1*^ΔE2/+^ mice. One possible explanation for this discrepancy is the presence of compensatory mechanisms in mice carrying the ΔE2 deletion. Although the SPTLC1 protein containing the ΔE2 deletion was expressed at very low levels as seen in homozygous mice (Fig S2A), the total level of the SPTLC1 protein was not significantly reduced in the *Sptlc1*^ΔE2/+^ mice. Alternatively, the impact of ALS-associated *SPTLC1* mutations on lipid metabolism may be more context-dependent than previously thought. Moreover, direct assessment of in vivo SPTLC1 enzymatic activity would provide important functional insight into the consequences of the ΔE2 mutation in mice. However, such measurements typically require metabolic flux approaches, such as administration of isotopically labeled serine, to quantify SPTLC1 pathway activity in vivo. These experiments are technically demanding and were beyond the scope of the current study. Instead, we assessed downstream functional output of the pathway using targeted sphingolipidomics across multiple tissues. These analyses did not reveal significant differences in sphingolipid species between heterozygous and control animals. Our in vivo data reflect steady-state metabolic outcomes rather than direct enzymatic activity. Studies measuring sphingolipid levels in patient-derived biofluids have produced conflicting results. Mohassel et al showed that c.58G>T patient plasma had increased levels of canonical sphingolipids, whereas another study from Johnson et al showed no change in two patients with the same mutation (3, 5). Given that lipid metabolism is influenced by multiple factors, including age, diet, and systemic metabolic regulation, our findings highlight the need for further investigation into the specific conditions under which the *SPTLC1* ΔE2 variant leads to dysregulated sphingolipid synthesis.

A key observation in our study is the embryonic lethality associated with the homozygous *Sptlc1* ΔE2 allele, albeit with incomplete penetrance. This early lethality precluded generation of a sufficiently powered homozygous cohort to perform experiments together with heterozygous or control mice. However, the *Sptlc1*^*ΔE2*^ knock-in allele is informative; although *Sptlc1* knockout mice were reported embryonic lethal, it is surprising that the ΔE2 allele is likewise nonviable when homozygous. It is possible that the residual, very low levels of the total SPTLC1 protein in these mice would result in aberrant sphingolipid homeostasis during development and would reflect a sharp threshold when transitioning to a lethal phenotype possibly because of very low levels of the SPTLC1 protein. Alternatively, unstable stoichiometry of SPT complex subunits (SPTLC1, SPTLC2, ORMDL3) may be irreparably perturbed in homozygotes. We were unable to reliably assess protein levels of SPT complexes, as commercially available antibodies against mouse SPTLC2, SPTLC3, and ORMDL3 proteins failed to yield specific signals. We instead expanded qPCR for key components of the SPT complex, including *Sptlc1*, *Sptlc2*, *Ormdl3*, as well as of small subunits *Sptssa* and *Sptssb* (Fig S2B). We acknowledge that mRNA levels do not capture potential posttranslational regulation of SPT-ORMDL protein complex stoichiometry, which remains a limitation of the current study. In a human patient with the exon 2 skipping variant, proteomics studies revealed a 50% reduction in total SPTLC1 levels in lymphoblast cells (20). However, no lipidomics analysis was performed in c.58G>T patient cells (20). Overall, although our data confirm that the ΔE2 allele produces a truncated protein in vivo, we acknowledge that further protein-level analyses will be necessary to definitively determine its impact on SPT complex assembly.

The first murine models of *Sptlc1* designed to understand the role of *SPTLC1* in a human disease context used an overexpression approach. Transgenic mice overexpressing SPTLC1-linked HSAN1 C133W mutation (using chicken beta-actin promoter with CMV enhancer elements and *Cricetulus griseus SPTLC1* cDNA) displayed age-dependent weight loss and mild sensory and motor impairments (13). Similarly, the overexpression of a fusion SPT (fSPT) construct consisting of three individual WT SPT subunits (SPTLC2, SPTSSA, and SPTLC1) resulted in neurological phenotypes in mice (21). Together, these studies demonstrate how increased enzyme activity levels can lead to severe phenotypes in rodents. Although informative, these models carry the caveat that the observed pathology may be due to transgene overexpression rather than being reflective of the human disease state that exhibits endogenous gene expression. Recently, a knock-in *Sptlc1*^C133W^ mouse model has been generated to address this for HSAN1 disease by the Burgess laboratory (12). The heterozygous mice were phenotypically normal but displayed the biochemical signatures of HSAN1, whereas homozygotes were lethal. It is not clear why homozygous *Sptlc1*^C133W^ mice were lethal. Nevertheless, these data suggest that sphingolipid synthesis is tightly controlled and critical for mouse development. Collectively, we demonstrate that *Sptlc1*^*ΔE2/+*^ knock-in mice do not recapitulate overt ALS features, such as motor function impairment, whereas homozygous mice die early. Additional genetic factors or environmental stressors may influence ALS disease progression. It remains unclear whether sufficient ΔE2 protein is available in vivo to incorporate into the SPT complex or interact with ORMDL proteins. Nonetheless, this model has been informative and will be useful for future mechanistic studies.

## Materials and Methods

### Generation of the *Sptlc1*^*ΔE2*^ mouse model

**Figure S3.**
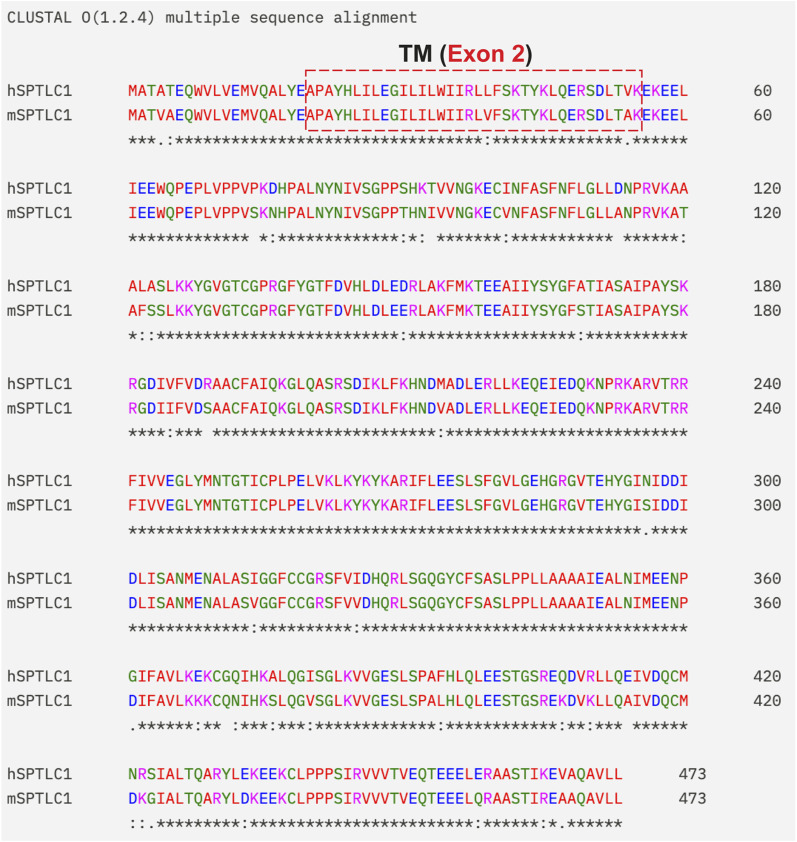
Overall organization of mouse and human SPTLC1. SPTLC1 is comprised of a transmembrane (TM) domain encoded by exon 2. Protein sequence alignment of human (NP_006406.1) and mouse (NP_033295.2), showing the position of the highly conserved TM domain (red box). The c.58G/T mutation induces the skipping of exon 2, resulting in a shortened sequence from residue 20 onward.

**Figure S4.**
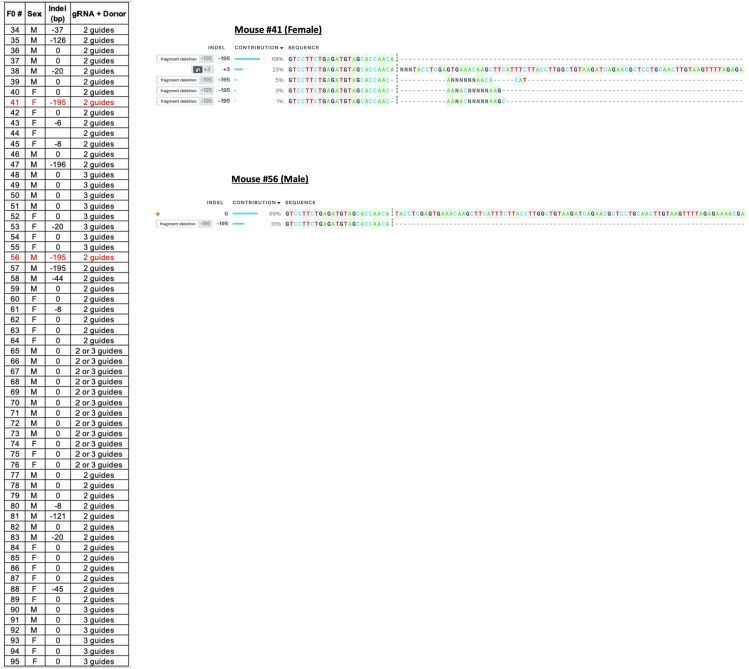
Analysis of sgRNAs that target the introns spanning *Sptlc1* exon 2 loci. Sequences of the 64 mice injected with Cas9, sgRNAs, and donor oligo show cleavage of the *Sptlc1* locus at the intron–exon–intron junction of exon 2. Base modification status is listed. Two positive mosaic founder (F0) lines #41 and #56 (in red) were selected as they displayed precise deletion of exon 2 with the highest % indel contribution (−195 bp) as confirmed by Synthego ICE in silico analysis.

**Figure S5.**
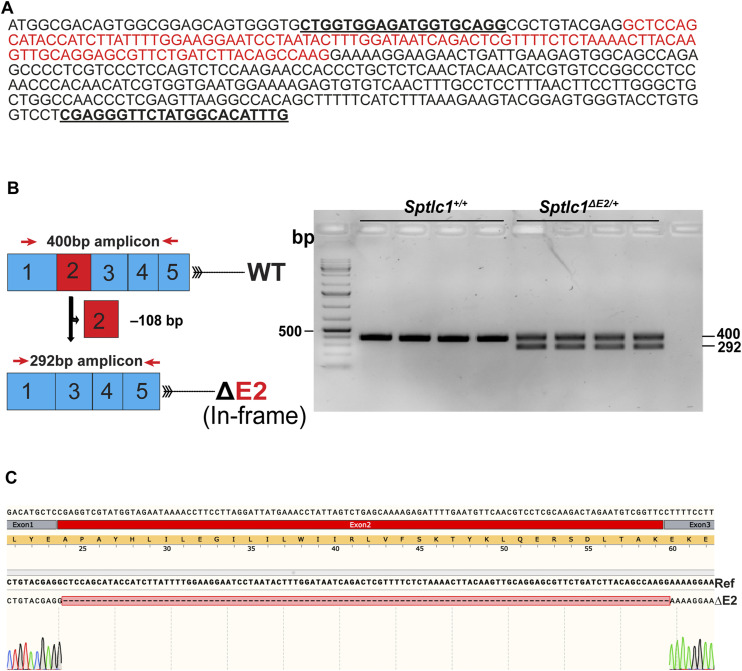
Characterization of the *Sptlc1^ΔE2/^*^+^ mouse F1 line. **(A)** cDNA sequence of the targeted *Sptlc1* locus, spanning exon 2 and surrounding regions. Primer sequences in exon 1 (forward) and exon 5 (reverse) are bold and underlined; exon 2 is indicated in red. **(B)** In-frame deletion of exon 2. Agarose gel electrophoresis of RT–PCR products to validate deletion of exon 2 using mRNA from mouse spinal cord tissues. RT–PCR primers were in exons 1 and 5 (red arrow), and the amplicon size is 400 bp for WT mice and 292 bp for ΔE2 F1 mice (n = 4, 4 mo old). **(C)** Sanger sequencing confirmed the precise deletion of only exon 2. Source data are available for this figure.

The *Sptlc1* knock-in mouse model, bearing an exon 2 deletion (ΔE2) in the *Sptlc1* gene (ENSMUSG00000021468), was generated via CRISPR/Cas9 genome editing. Both mouse and human SPTLC1 have high sequence conservation (Fig S3). Zygotes from C57BL/6J (RRID: IMSR_JAX:000664) mice were microinjected with single-guide RNAs (sgRNAs) targeting the introns flanking exon 2 (GCA​GAC​TTT​GAA​GCA​GCT​TC, TTG​TTT​CAC​TCG​AGG​TAT​GT, GAA​AGG​CTT​GAG​AGC​CAT​TG), Cas9 protein (Synthego), and a 120-bp donor template (GTG​TGG​TAA​GAA​TGT​GAA​GGT​CAG GAG​AGG​CAG​CAC​TGT​GGT​GGG​CTC​CAT​TTC​CCA​GCA​TGT​TGG​TGC​TAC​ATC​TCA​GAA​GGA​CTC​AAA​CTG​GAC​CCT​GTA​GTG​TGT​CAA​AAT​GCT​TCA). These zygotes were subsequently implanted into surrogate female mice with the assistance of the Emory Mouse Transgenic and Gene Targeting Core. All mouse experimental protocols were approved by the Institutional Animal Care and Use Committee at Emory University. Mice were housed on a 12/12-h light/dark cycle with access to standard mouse chow (Cat#5001; LabDiet) and water ad libitum. Two founder mice (F_0_) with a 195-bp deletion encompassing exon 2 were identified and selected for colony establishment (Fig S4). To minimize mosaicism and potential off-target events, F_0_ mice were backcrossed with WT C57BL/6J mice for two generations before generating the experimental cohorts described. Genotyping of genomic DNA from tail biopsies was performed using the following primers: GCT​GAC​AGT​GTT​GGG​GTT​TT and ACT​TCG​TAC​CCC​CAG​CTC​TC. The in-frame deletion of exon 2 was confirmed by polymerase chain reaction (PCR) using cDNA from heterozygous mice with primers spanning exon 1 to exon 5 using the following primers: CTG​GTG​GAG​ATG​GTG​CAG​G and CAA​ATG​TGC​CAT​AGA​ACC​CTC (Fig S5A and B). Finally, the exon 2 deletion was validated by Sanger sequencing (Fig S5C).

### Mouse behavioral testing

Experimenters were blinded to mouse genotype for all experiments. *Sptlc1*^*+/+*^ and *Sptlc1*^*ΔE2/+*^ mice were used in all behavioral tests. Mice were habituated to the testing room for 30–45 min (unless otherwise specified) before the start of each behavioral experiment to minimize stress-induced variability.

### Grip strength assessment

Forelimb and hindlimb grip strength were assessed using a Grip Strength Meter (Ugo Basile). The apparatus consisted of a base plate and a T-shaped grasping bar with adjustable height, attached to a force transducer connected to a peak amplifier. Before each measurement, the gauge was reset to 0 g after stabilization to ensure accuracy. The mouse was placed over the base plate in front of the bar, and its tail was slowly pulled back by the experimenter until the animal released its grip. The maximal tension recorded at the time of release was used as the grip strength.

### Rotarod test

Locomotor function was assessed using a rotarod apparatus on a five-lane rotating drum (ENV-574M, Med Associates) with a 1.5875 cm radius and gradually increasing speed protocol. The speed ramped from 5 to 40 rpm over 5 min, with a maximum trial duration of 10 min. Mice underwent three trials per day for three consecutive days, with a 60-min acclimation period in the testing room before the first trial each day. During each trial, the latency to fall from the rotating rod or to make two consecutive complete rotations while clinging to the rod was recorded. Mice were allowed to rest for at least 15 min between each trial. The average latency across the three trials was calculated as an indicator of locomotor function. Between trials, the rotarod apparatus was cleaned with 70% ethanol to maintain consistent testing conditions.

### CMAP recording

Electrophysiological measurements of CMAP were performed using needle electrodes, a minimally invasive and highly sensitive method for monitoring neuromuscular function, as previously described (17). Briefly, mice were anesthetized with isoflurane (3–5% for induction, 2–3% for maintenance) to ensure immobilization and minimize discomfort. Monopolar needle electrodes (Rhythmlink) were placed in the left hindlimb for motor nerve stimulation and CMAP recordings. An active needle electrode was inserted in the left gastrocnemius–soleus muscle, whereas the reference was inserted in the ipsilateral tendinous heel. The ground electrode was inserted subcutaneously in the upper midback of the mouse. The cathode and anode needle electrodes were inserted on either side of the left sciatic nerve at the proximal thigh. The left sciatic nerve was stimulated with a 0.1-ms pulse duration and an intensity 1–10 mA using a portable electrodiagnostic system (Cadwell Sierra Summit). CMAP peak-to-peak amplitudes were recorded three times while increasing current intensity and adjusting the active recording electrode to obtain a supramaximal CMAP. The highest amplitude among the three recordings was used for data analysis.

### Lipidomics

Lipids were extracted from frozen tissues (10 mg) with 500 μl of methanol containing 200 pmoles of the following internal standards: D_7_-sphinganine (d18:0), D_7_-sphingosine (d18:1), dihydroceramide (d18:0/12:0), and ceramide (d18:1/12:0). Samples were incubated on a shaker incubator at 37°C and 1,400 rpm for 1 h. Lipids were hydrolyzed overnight at 65°C and subsequently re-extracted with chloroform as described previously (12). Hydrolyzed lipids were dried under a stream of nitrogen gas and resuspended in 200 μl (tissue) reconstitution buffer (70% methanol, 10 mM ammonium acetate, pH 8.5). LCBs were separated via a reverse-phase C18 column (Uptispere 120 Å, 5 μm, 125 × 2 mm; Interchim) connected to QTRAP 6500+ LC-MS/MS System (SCIEX). LCBs were quantified by normalization to the internal standards and tissue protein content. For protein normalization, tissue pellets were homogenized in 8 M urea using a Precellys 24 tissue homogenizer (Bertin Technologies) post-extraction. Protein concentration was determined using the Bradford assay.

### Immunofluorescence staining

Mouse brain and spinal cord tissues after dissection were immersed and fixed in 4% PFA for 24 h at 4°C to ensure proper penetration to maintain morphological integrity and cryoprotected in 30% sucrose (Sigma-Aldrich) for 48 h. The tissues were embedded in optimal cutting temperature compound and sectioned at 20-μm thickness. Sections were mounted on Superfrost Plus glass slides (VWR) and stored at −20°C. Sections were permeabilized with 0.2% Triton X-100 (Sigma-Aldrich) for 10 min and blocked in 5% BSA for 1 h at room temperature. Primary antibodies diluted in blocking solution were applied, and sections were incubated for 24 h at 4°C. The following primary antibodies were used: anti-NeuN antibody (guinea pig polyclonal, 266004, 1:1,000; Synaptic Systems), anti-IBA1 antibody (rabbit polyclonal, 01919741, 1:1,000; Wako), and anti-GFAP antibody (rabbit polyclonal, ab7260, 1:500; Abcam). Sections were rinsed with 1× PBS and then incubated with a secondary antibody Alexa Fluor 488 donkey anti-rabbit IgG (H+L), 1:500 (Thermo Fisher Scientific), Cy5 AffiniPure Donkey Anti-Guinea Pig IgG (H+L), 1:500 (Jackson ImmunoResearch), and DAPI (D6210, 1:1,000; Millipore) for 24 h at 4°C. Sections were rinsed with PBS and mounted with ProLong Gold Antifade Mountant (P36930; Thermo Fisher Scientific). For quantification of GFAP, IBA1, and NEUN stainings, images were taken at 60× and 20× for brain and spinal cord, respectively. Images were stitched and analyzed throughout each hippocampal region of mouse sagittal brain sections and whole spinal cord sections to determine the average fluorescence intensity (A.U.) across the region of interest. Fluorescence images were acquired using a BZ-X810 fluorescence microscope (Keyence). Images shown in Figs 4 and S1 were obtained using a 20× objective lens. For samples exceeding a single field of view, multiple overlapping images were captured and automatically stitched using BZ-X Analyzer software (Keyence) to generate composite images. Higher magnification images were acquired using a 60× objective lens for Fig S1. The fluorescence filter sets used were as follows: DAPI, excitation 360/40 nm and emission 460/50 nm; GFP/FITC, excitation 470/40 nm and emission 525/50 nm; and TRITC/Alexa Fluor 594, excitation 545/25 nm and emission 605/70 nm. Images were analyzed by ImageJ software.

### Western blotting

Tissue extracts were prepared using RIPA lysis buffer (pH 7.4; bioWORLD) supplemented with Halt protease and phosphatase inhibitor cocktail (Thermo Fisher Scientific). DNA shearing was performed to reduce sample viscosity. After centrifugation, protein concentrations were determined using BCA Protein Assay Reagent (Pierce). Twenty micrograms of protein was resolved on 4–20% precast polyacrylamide gels (Bio-Rad) and transferred to nitrocellulose membranes (Bio-Rad). Membranes were blocked and incubated overnight at 4°C with the following primary antibodies: mouse anti-SPTLC1 (1:2,000; BD Biosciences), rabbit anti-β actin (1:2,000; GeneTex), and rabbit anti-GAPDH (1:2,000; Cell Signaling Technology). Membranes were then incubated for 1 h at room temperature with secondary antibodies: HRP-conjugated secondary antibodies (ABclonal) or IRDye secondary antibodies (LI-COR). For detection, SuperSignal West Pico (Pierce) was used to visualize peroxidase activity. Protein molecular masses were determined by comparison with protein standards (Thermo Fisher Scientific). Band intensities were quantified using ImageJ software and normalized to GAPDH or β-actin as loading controls.

### RT–qPCR analysis

Total RNA was prepared from mouse tissues using TRIzol reagent (Invitrogen) and purified with the Quick-RNA Miniprep kit (Zymo Research) according to the manufacturer’s instructions. Complementary DNA (cDNA) was generated using High-Capacity cDNA Reverse Transcription Kit (Thermo Fisher Scientific) following the manufacturer’s protocol. PCR amplification was performed under the following conditions: 95°C for 30 s, (95°C for 30 s, 57°C for 30 s, 72°C for 1 min) for 35 cycles, followed by an extension stage of 72°C for 5 min and a 10°C hold. To quantify relative mRNA expression, RT-qPCR was performed using TaqMan gene expression assays (FAM-labeled; Thermo Fisher Scientific) on a QuantStudio 6 Flex system (Applied Biosystems). The following TaqMan assay probes were used: *Sptlc1* (Mm00447343_m1, Exon 3–4), *Sptlc2* (Mm00448871_m1, Exon 4–5), *Ormdl3* (Mm00787910_sH, Exon 4–4), *Sptssa* (Mm01267361_g1, Exon 1–2), *Sptssb* (Mm01952615_u1, Exon 4–4), *Iba1* (Mm00479862_g1, Exon 4–5), *Gfap* (Mm01253033_m1, Exon 6–7). Relative RNA expression was normalized to *Gapdh* (Mm99999915_g1, Exon 2–3).

### Statistical analysis

All statistical analyses and figures were prepared using GraphPad Prism (version 9). Data are expressed as the mean ± SD or mean ± SEM as indicated in the respective figure legends. *t* test was used for two-group comparisons, and one-way or two-way ANOVA was used for multiple-group comparisons unless otherwise specified in the figure legends. A *P*-value less than 0.05 was considered statistically significant.

## Supplementary Material

Reviewer comments

## Data Availability

Requests for additional data supporting results should be directed to and will be fulfilled by the corresponding author. Emory University Animal Care Committee provided official ethics board approval for this study. Tissues used in this study were collected from mice maintained and handled in accordance with the approved protocols.
