# Supplementary figures and images for: Deletion of exon 2 in ALS-linked Sptlc1 causes lethality in homozygous mice but not in heterozygotes
Source: Life Sci Alliance. 2026 Jul 2;9(9):e202503605. doi: 10.26508/lsa.202503605 (PMC13329131; doi:10.26508/lsa.202503605)

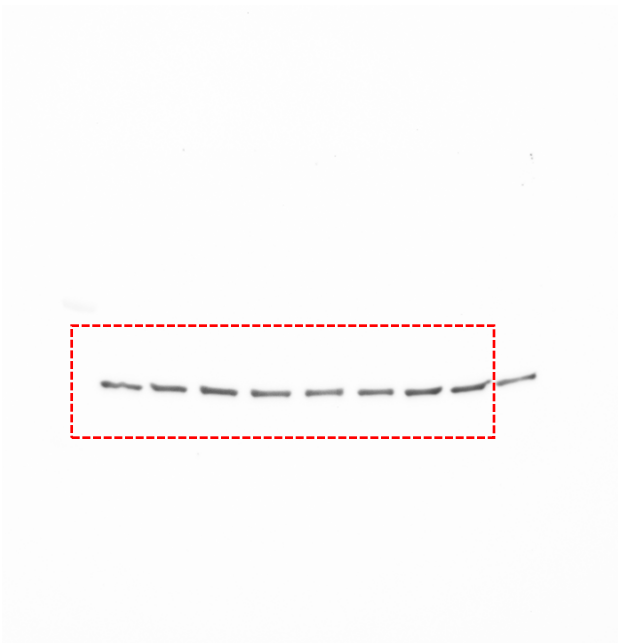

Supplement: Supplementary file 1 [file LSA-2025-03605_SdataF1.pdf]

Fig S2A (uncropped)

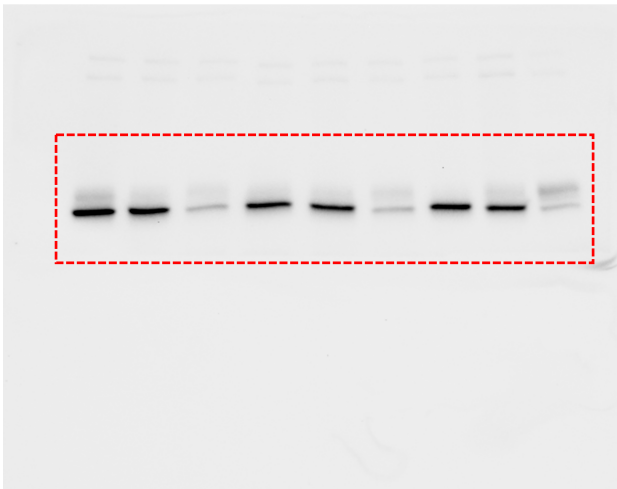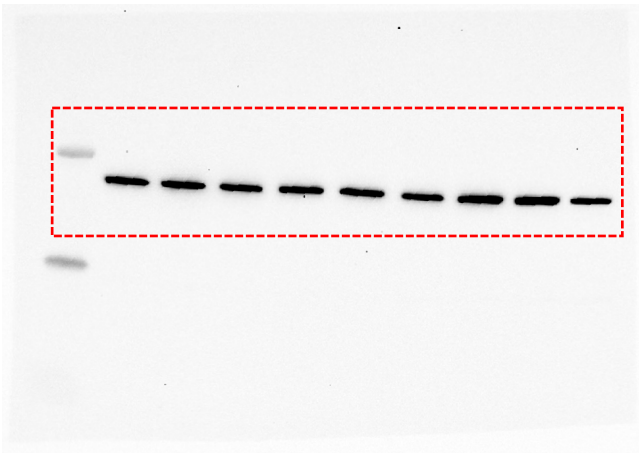

Supplement: Supplementary file 2 [file LSA-2025-03605_SdataFS2.pdf]

**Fig S5B (uncropped)**

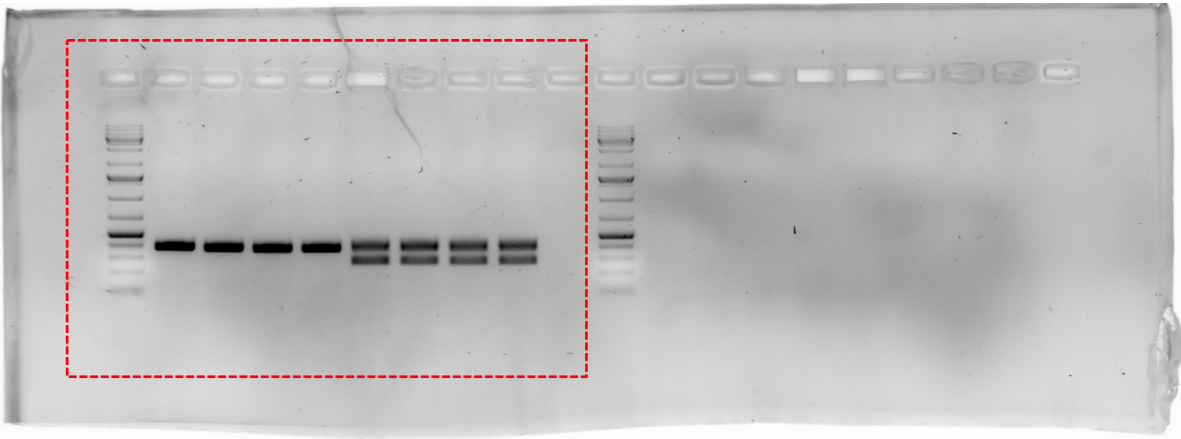

Supplement: Supplementary file 3 [file LSA-2025-03605_SdataFS5.pdf]
