# Supplementary material for: Deletion of exon 2 in ALS-linked Sptlc1 causes lethality in homozygous mice but not in heterozygotes
Source: Life Sci Alliance. 2026 Jul 2;9(9):e202503605. doi: 10.26508/lsa.202503605 (PMC13329131; doi:10.26508/lsa.202503605)
Supplement: Reviewer comments [file LSA-2025-03605_review_history.pdf]

Life Science Alliance

Deletion of exon 2 in ALS-linked Sptlc1 causes lethality in homozygous mice but not in heterozygotes

Devesh C Pant, Museer A. Lone, Janani Parameswaran, Fuying Ma, Nicole Ziak, Prisha Dutta, Zitong Wang, Daniel Pun, Sumit Verma, Thorsten Hornemann and Jie Jiang

DOI: https://doi.org/10.26508/lsa.202503605

Corresponding author(s): Dr. Devesh C Pant (Emory University)

Review Timeline:

Submission Date:	2025-12-17
Editorial Decision:	2025-12-17
Revision Received:	2026-01-09
Editorial Decision:	2026-03-09
Revision Received:	2026-05-11
Editorial Decision:	2026-06-16
Revision Received:	2026-06-16
Accepted:	2026-06-19

Scientific Editor: Sarita Hebbar

Transaction Report:

(Note: With the exception of the correction of typographical or spelling errors that could be a source of ambiguity, letters and reports are not edited. The original formatting of letters and referee reports may not be reflected in this compilation.)

No full Peer Review Process File is available with this article, as the authors have chosen not to make the review process public in this case.

December 17, 2025

Re: Life Science Alliance manuscript #LSA-2025-03605P

Devesh C Pant
Emory University
Department of Cell Biology
615 Michael St, Rm 467
Atlanta, GA 30329

Dear Dr. Pant,

Thank you for submitting your manuscript entitled "Deletion of Sptlc1 transmembrane domain hotspot for early-onset ALS causes early lethality in homozygous mice and no phenotypic abnormalities in heterozygous mice" to Life Science Alliance.

The editorial team has discussed your inquiry and we feel this work fits the scope of LSA. We are pleased to invite submission of this manuscript for full editorial evaluation. Please use the link below to submit your manuscript. When doing so please ensure to include all authors and their contact information. Feel free to contact us if you have any further questions concerning this work.

To upload your manuscript, please use: https://lsa.msubmit.net/cgi-bin/main.plex
If you have problems accessing this, please email: contact@life-science-alliance.org

Thank you for thinking of LSA when considering potential outlets for your manuscript.

Sincerely,

Sarita Hebbar, PhD
Scientific Editor
Life Science Alliance
http://www.lsajournal.org

March 10, 2026

Re: Life Science Alliance manuscript #LSA-2025-03605

Dr. Devesh C Pant
Emory University
Department of Cell Biology
615 Michael St, Rm 467
Atlanta, GA 30329

Dear Dr. Pant,

Thank you for submitting your manuscript entitled "ALS-linked Sptlc1 exon 2 deletion causes lethality in homozygous mice but not in heterozygotes" to Life Science Alliance. Your manuscript was evaluated by three expert reviewers whose comments are appended below.

When submitting the revision, please include a letter addressing the reviewers' comments point by point. While a rebuttal must respond to all points in some form, additional experiments to resolve these points, other than indicated above, are not required.

I would be happy to discuss the revision in more detail via email or phone/videoconferencing. Please let me know which option you prefer, if any.

To upload the revised version of your manuscript, please log in to your account: https://lsa.msubmit.net/cgi-bin/main.plex

You will be guided to complete the submission of your revised manuscript and to fill in all necessary information. Please get in touch in case you do not know or remember your login name.

The typical timeframe for revisions is three months. Please note that papers are generally considered through only one revision cycle, so strong support from the referees on the revised version is needed for acceptance.

While you are revising your manuscript, please also attend to the below editorial points to help expedite the publication of your manuscript. Please direct any editorial questions to the journal office.

Thank you for this interesting contribution to Life Science Alliance. We hope that the comments below will prove constructive as your work progresses, and we are looking forward to receiving your revised manuscript.

Sincerely,

Sarita Hebbar, PhD
Scientific Editor
Life Science Alliance
http://www.lsajournal.org

A. THESE ITEMS ARE REQUIRED FOR REVISIONS

-- A letter addressing the reviewers' comments point by point.

-- An editable version of the final text (.DOC or .DOCX) is needed for copyediting (no PDFs).

-- High-resolution figure, supplementary figure and video files uploaded as individual files: See our detailed guidelines

for preparing your production-ready images, https://www.life-science-alliance.org/authors

-- Summary blurb (enter in submission system): A short text summarizing in a single sentence the study (max. 200 characters including spaces). This text is used in conjunction with the titles of papers, hence should be informative and complementary to the title and running title. It should describe the context and significance of the findings for a general readership; it should be written in the present tense and refer to the work in the third person. Author names should not be mentioned.

-- By submitting a revision, you attest that you are aware of our payment policies found here: https://www.life-science-alliance.org/copyright-license-fee

B. MANUSCRIPT ORGANIZATION AND FORMATTING:

Full guidelines are available on our Instructions for Authors page, https://www.life-science-alliance.org/authors

We encourage our authors to provide original source data, particularly uncropped/-processed electrophoretic blots and spreadsheets for the main figures of the manuscript. If you would like to add source data, we would welcome one PDF/Excel-file per figure for this information. These files will be linked as supplementary "Source Data" files.

IMPORTANT: It is Life Science Alliance policy that if requested, original data images must be made available. Failure to provide original images upon request will result in unavoidable delays in publication. Please ensure that you have access to all original microscopy and blot data images before submitting your revision.

June 16, 2026

RE: Life Science Alliance Manuscript #LSA-2025-03605R

Dr. Devesh C Pant
Emory University
Department of Cell Biology
615 Michael St, Rm 467
Atlanta, GA 30329

Dear Dr. Pant,

Thank you for submitting your revised manuscript entitled "ALS-linked Sptlc1 exon 2 deletion causes lethality in homozygous mice but not in heterozygotes".

Your manuscript was evaluated by two of the original reviewers whose comments are included below. In line with the reviewers' overall evaluation, we would be happy to publish your paper in Life Science Alliance pending final revisions necessary to meet our formatting guidelines.

MANUSCRIPT ORGANIZATION AND FORMATTING:

To avoid unnecessary delays in the acceptance and publication of your paper, please read the following information carefully. Full guidelines are available on our Instructions for Authors page, https://www.life-science-alliance.org/authors

Submission of a paper that does not conform to Life Science Alliance guidelines will delay the acceptance of your manuscript.

-We encourage you to change the ms title to, "Deletion of exon 2 in ALS-linked Sptlc1 causes lethality in homozygous mice but not in heterozygotes".
-We encourage you to check the cropped version of the actin blot in Figure 1D as it may inadvertently appear as a manipulated image.
-Kindly include molecular weight markers for blots shown in Figures 1D and S5.
-Please provide the sequence for primers indicated in Figure S3B.
-Kindly expand on the imaging section to provide details on objectives and excitation and emission filters for fluorescence imaging.
-Please provide a scale bar and size information for higher magnification images shown in Figure S4 (GFPA).
-Please check accuracy for axes titles for Figure 3C.
-Thank you for providing a statement on data availability. We request you to follow our guidelines on data availability (https://www.life-science-alliance.org/editorial-policies#data-sharing)
-LSA does not permit citation of "data not shown," "manuscript in preparation," "manuscript submitted," etc., in any section of the manuscript. For the statement in line 304, either provide the data to support the claim or remove the claim completely.
-Please rename "Declaration of competing interest" to Conflict of Interest".
-Please add an Author Contributions section to your main manuscript text.
-Please add your main and supplementary legends to the main manuscript text after the references section.
-Please remove legends from the Supplementary figures.
-Please add callouts for Figures 4D, E; 5A-E; S3A-C; S4A-C and S5A, B to your main manuscript text.
-Please remind the 2ndary Corresponding Author to add ORCID ID to their account - they should have received instructions on how to do so.
-Please be sure that the authorship listing and order are correct and match between the system and the manuscript file.
-Please consult our manuscript preparation guidelines https://www.life-science-alliance.org/manuscript-prep and make sure your manuscript sections are in the correct order.

-Please be sure that the authorship listing and order is correct

We encourage our authors to provide original source data, particularly uncropped/-processed electrophoretic blots and spreadsheets for the main figures of the manuscript. If you would like to add source data, we would welcome one PDF/Excel-file per figure for this information. These files will be linked as supplementary "Source Data" files.

It is Life Science Alliance policy that if requested, original data images must be made available to the editors. Failure to provide original images upon request will result in unavoidable delays in publication. Please ensure that you have access to all original data images prior to final submission.

If you are planning a press release on your work, please inform us immediately to allow informing our production team and scheduling a release date.

We welcome submissions of potential cover images for the issue of LSA in which your work would appear. If you have high quality images associated with this work, please feel free to email these, with a caption, to the journal office.

LSA encourages authors to provide a 30-60 second video where the study is briefly explained. These videos will be appear embedded with the manuscript online at Life Science Alliance, and on social media to promote the published paper and authors (for examples, see https://docs.google.com/document/d/1-UWCfbE4pGcDdcgzcmiuJl2XMBJnxKYeqRvLLrLSo8s/edit?usp=sharing). Corresponding or first-authors are welcome to submit the video. Please submit only one video per manuscript. The video can be emailed to contact@life-science-alliance.org

FINAL FILES:

To upload the final version of your manuscript, please log in to your account: https://lsa.msubmit.net/cgi-bin/main.plex
You will be guided to complete the submission of your revised manuscript and to fill in all necessary information. Please get in touch in case you do not know or remember your login name.

The following items are required for acceptance.

-- An editable version of the final text (.DOC or .DOCX) is needed for copyediting (no PDFs).

-- High-resolution figure, supplementary figure and video files uploaded as individual files: See our detailed guidelines for preparing your production-ready images, https://www.life-science-alliance.org/authors

-- Summary blurb (enter in submission system): A short text summarizing in a single sentence the study (max. 200 characters including spaces). This text is used in conjunction with the titles of papers, hence should be informative and complementary to the title. It should describe the context and significance of the findings for a general readership; it should be written in the present tense and refer to the work in the third person. Author names should not be mentioned.

The license to publish form must be signed before your manuscript can be sent to production. A link to the license to publish form will be available to the corresponding author only. Please take a moment to check your funder requirements.

Reviews, decision letters, and point-by-point responses associated with peer-review at Life Science Alliance will be published, alongside the manuscript. If you do want to opt out of having the reviewer reports and your point-by-point responses displayed, please let us know immediately.

Thank you for your attention to these final processing requirements. Please revise and format the manuscript and upload materials as soon as you are able.

Thank you for this interesting contribution to the literature. We look forward to publishing your paper in Life Science Alliance.

Sincerely,

Sarita Hebbar, PhD
Scientific Editor
Life Science Alliance
http://www.lsajournal.orgyour revision.***

June 19, 2026

RE: Life Science Alliance Manuscript #LSA-2025-03605RR

Dr. Devesh C Pant
Emory University
Department of Cell Biology
615 Michael St, Rm 467
Atlanta, GA 30329

Dear Dr. Pant,

Thank you for submitting your Research Article entitled "Deletion of exon 2 in ALS-linked Sptlc1 causes lethality in homozygous mice but not in heterozygotes". It is a pleasure to let you know that your manuscript is now accepted for publication in Life Science Alliance. Congratulations on this interesting work.

The final published version of your manuscript will be deposited by us to PubMed Central upon publication.

Your manuscript will now progress through copyediting and proofing. It is journal policy that authors provide original data upon request.

Your article will publish open access upon publication under a CC-BY license.

Reviews, decision letters, and point-by-point responses associated with peer-review at Life Science Alliance will be published, alongside the manuscript. If you do want to opt out of having the reviewer reports and your point-by-point responses displayed, please let us know immediately.

IMPORTANT: If you will be unreachable at any time, please provide us with the email address of an alternate author. Failure to respond to routine queries may lead to unavoidable delays in publication.

Scheduling details will be available from our production department. You will receive proofs shortly before the publication date. Only essential corrections can be made at the proof stage so if there are any minor final changes you wish to make to the manuscript, please let the journal office know now.

DISTRIBUTION OF MATERIALS:
Authors are required to distribute freely any materials used in experiments published in Life Science Alliance. Authors are encouraged to deposit materials used in their studies to the appropriate repositories for distribution to researchers.

You can contact the journal office with any questions, contact@life-science-alliance.org

Again, congratulations on a very nice paper. I hope you found the review process to be constructive and are pleased with how the manuscript was handled editorially. We look forward to future exciting submissions from your lab.

Sincerely,

Sarita Hebbar, PhD
Scientific Editor
Life Science Alliance
http://www.lsajournal.org
